# Smoking is an independent but not a causal risk factor for moderate to severe psoriasis: A Mendelian randomization study of 105,912 individuals

**DOI:** 10.3389/fimmu.2023.1119144

**Published:** 2023-02-22

**Authors:** Charlotte Näslund-Koch, Signe Vedel-Krogh, Stig Egil Bojesen, Lone Skov

**Affiliations:** ^1^ Department of Dermatology and Allergy, Copenhagen University Hospital - Herlev and Gentofte, Copenhagen, Denmark; ^2^ Department of Clinical Medicine, University of Copenhagen, Copenhagen, Denmark; ^3^ Department of Clinical Biochemistry, Copenhagen University Hospital - Herlev and Gentofte, Copenhagen, Denmark; ^4^ Copenhagen General Population Study, Copenhagen University Hospital - Herlev and Gentofte, Copenhagen, Denmark

**Keywords:** psoriatic disease, psoriasis vulgaris, tobacco consumption, cigarettes, observational, genetic, epidemiology, causality

## Abstract

**Background:**

Smoking is strongly associated with higher risk of psoriasis in several observational studies; however, whether this association is causal or can be explained by confounding or reverse causation is not fully understood. Randomized controlled trials are the gold standard when examining causality; however, when this method is not feasible, the Mendelian randomization design is an alternative. Herein genetic variants can be used as robust proxies for modifiable exposures and thereby avoiding confounding and reverse causation.

In this study, we hypothesized that smoking is an independent and causal risk factor for psoriasis and tested this using a Mendelian randomization design.

**Methods:**

We used data from the Copenhagen General Population Study including 105,912 individuals with full information on lifestyle factors, biochemistry, and genotype data. In total, 1,240 cases of moderate to severe psoriasis were included to investigate the association between smoking and psoriasis. To assess causality of the association, we used the genetic variant *CHRNA3* rs1051730, where the T-allele is strongly associated with high lifelong cumulative smoking, as a proxy for smoking.

**Results:**

In observational analyses, the multivariable adjusted hazard ratio of developing moderate to severe psoriasis was 1.64 (95% confidence interval: 1.35-2.00) in ever smokers with ≤ 20 pack-years and 2.23 (1.82-2.73) in ever smokers with > 20 pack-years compared to never smokers. In genetic analyses, the odds ratio of developing moderate to severe psoriasis was 1.05 (0.95-1.16) per *CHRNA3* rs10511730 T-allele in ever smokers.

**Conclusion:**

Smoking was an independent risk factor for moderate to severe psoriasis in observational analyses. However, using a genetic variant as a robust proxy for smoking, we did not find this association to be causal.

## 1 Introduction

**Figure 1:**
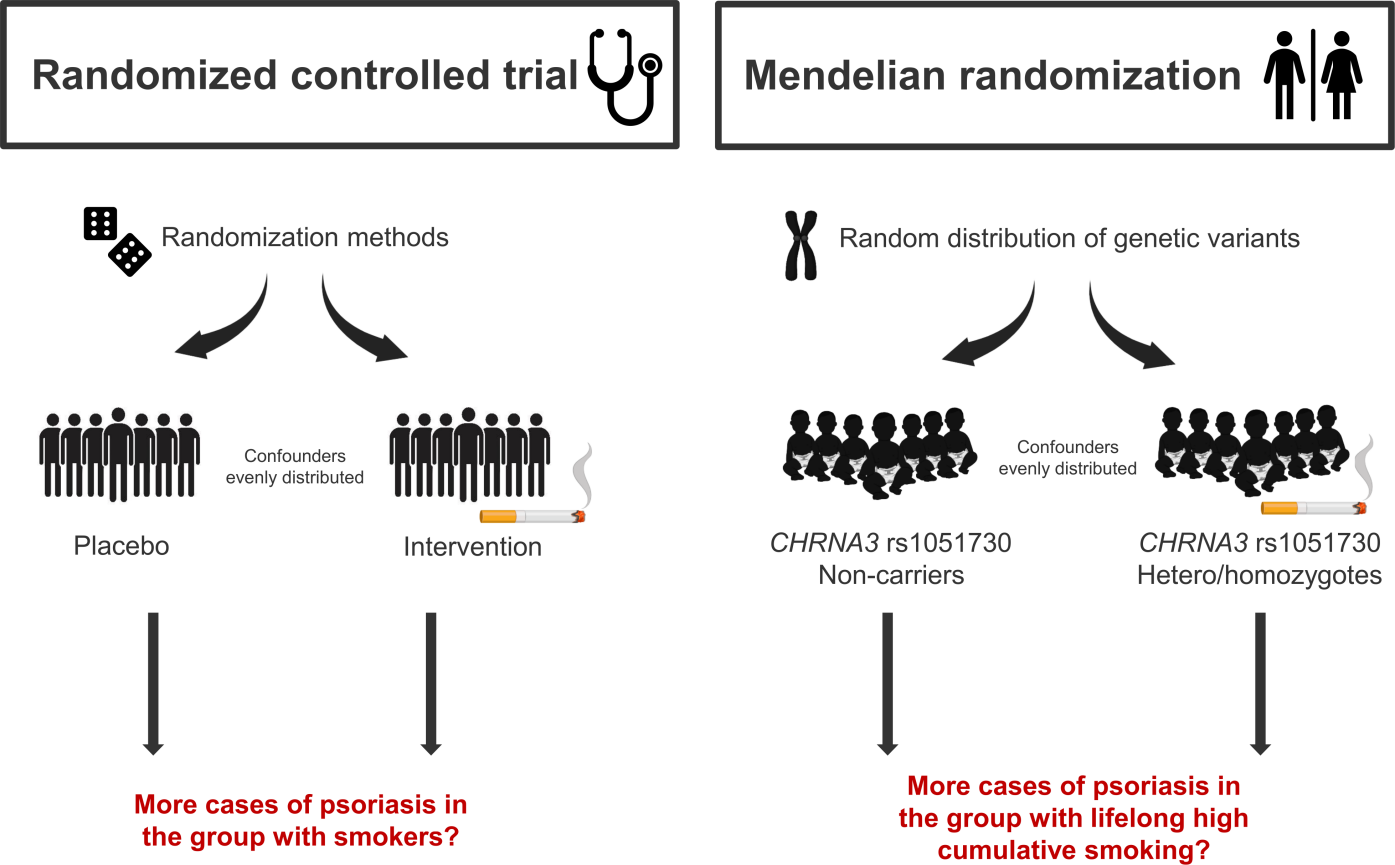
Randomized controlled trial vs. Mendelian randomization. A Mendelian randomization study takes advantages of the natural distribution of genetic variants during conception, and therefore mimics the randomized controlled trial. Using the genetic variant *CHRNA3* rs1051730, where the T-allele is strongly associated with lifelong high cumulative smoking, as a proxy for smoking, confounding factors are evenly distributed and since genes are present from birth they are not influenced by reverse causation.

Psoriasis affects 125 million people worldwide and is a chronic inflammatory autoimmune skin disease (1) associated with high risk of several comorbidities such as metabolic syndrome and cardiovascular disease (2); comorbidities that are closely related to smoking (3). Globally, 1.14 billion people smoke and tobacco consumption is of huge concern to public health (4). Patients with psoriasis are more likely to be smokers (5) and in observational studies, smoking is associated with higher risk of developing psoriasis (6–10). Furthermore, evidence suggests that prenatal tobacco exposure is associated with psoriasis in childhood (11). Taken together, evidence suggest that smoking is a strong risk factor for psoriasis. However, whether these findings reflect an actual causal association between smoking and psoriasis or can be explained by confounding or reverse causation is unclear. The gold standard to investigate causal associations is the randomized controlled trial. However, some exposures (e.g., smoking) cannot be examined in this type of study. Mendelian randomization (MR) is an epidemiological method which serves as a good alternative (12, 13). Based on Mendel´s law of inheritance, genetic variants can be used as robust proxies for modifiable exposures (in this case smoking) and causal associations can therefore be explored in an observational setting. Normally, confounding and reverse causation are intrinsic limitations in observational studies; however, in MR studies genetic variants are used as proxies for modifiable exposures. Germline genetic variants are randomly distributed during conception and are therefore not associated with confounders. Furthermore, genes are not influenced by reverse causation because they are present from birth (12). The gene *CHRNA3* is found in the nicotinic acetylcholine receptor gene cluster on chromosome 15 (14, 15). The T-allele of this genetic variant is strongly associated with nicotine dependence and cumulative smoking (15), and has therefore been used as an instrument for smoking in several previous MR studies (16–20). Using this genetic variant as a proxy for high lifelong cumulative smoking, it is achievable to elucidate the causal association between smoking and psoriasis (**Figure 1**). In this study, we hypothesized that smoking is an independent and causal risk factor for psoriasis and tested this using observational and genetic data from more than 100,000 individuals from the adult Danish general population.

## 2 Methods

### 2.1 Study population

The Copenhagen General Population Study is a prospective general population study initiated in 2003 (21). Individuals between 20 and 100 years of age who lived in greater Copenhagen were randomly invited, using the unique Danish identification number assigned at birth (22). Participation rate was 43%. At baseline visit, participants answered a questionnaire about health status and lifestyle factors and were physical examined. Subsequently, they had blood drawn for biochemical and genetic analyzes. We included 105,912 individuals with complete information on smoking, of whom 104,794 were genotyped for *CHRNA3*. We only included individuals of Danish descent in the present study to avoid population stratification, which can be an issue in MR studies when examining individuals from different populations (12).

All participants gave written informed consent. The study was conducted according to the Declaration of Helsinki and approved by the Danish ethical committee (H-KF-01-144/01).

### 2.2 Moderate to severe psoriasis

Individuals with psoriasis were identified using the World Health Organization International Classification of Diseases (WHO ICD) codes for psoriasis: ICD-8 696.09, 696.10, 696.19, and ICD-10 L40, compatible with moderate to severe psoriasis (23). In sensitivity analyses, we used a more limited definition of psoriasis (ICD-8 code 696.19; ICD-10 codes L40.0, L40.9). Information on diagnosis was collected from January 1977 to December 2018 from the Danish National Patient Registry (24).

### 2.3 Smoking and genotyping

Information on smoking was self-reported at the time of enrollment and participants were grouped as never, former, or current smokers. Former and current smokers also reported information on duration of tobacco consumption, and amount of consumed tobacco (cigarettes, cheroots, cigars, pipe tobacco), allowing us to calculate cumulative tobacco consumption in pack-years, i.e., one pack-year = 20 cigarettes (or equivalent tobacco) smoked daily for a year.

Genotyping was blinded to smoking status and psoriasis. DNA was isolated from whole blood and stored at −45°C. *CHRNA3* rs1051730 was genotyped using TaqMan assays (Applied Biosystems, Foster City, CA, USA) and verified by DNA sequencing. Reruns were performed twice, and call rates were >99.8%.

### 2.4 Covariates

Alcohol consumption, physical activity, and educational level were self-reported. High alcohol consumption was defined as alcohol consumption >168 g/week for men and >84 g/week for women. Low physical activity was defined as less than 2 hours of light physical activity per week in leisure time. Less than 3 years of education after mandatory primary school was considered a low education level. Body mass index (BMI) was calculated from measured weight and height (kg/m^2^). BMI was categorized according to WHO in groups <18.5, 18.5-24.9, 25-29.9, 30-34.9, 35-39.9, and ≥40 (25). Blood pressure was measured during the physical examination and hypertension was systolic blood pressure ≥140 mmHg, and/or diastolic blood pressure ≥90 mmHg, or self-reported use of antihypertensive treatment. Dyslipidemia was defined as total cholesterol ≥5 mmol/L (190 mg/dL), non-fasting low-density lipoprotein (LDL) cholesterol ≥3 mmol/L (115 mg/dL), or self-reported use of statins. Type 2 diabetes mellitus was defined as non-fasting plasma glucose >11 mmol/L (198 mg/dL), a registered diabetes diagnosis prior to baseline using ICD-8 250 and ICD-10 E11, E13, and E14, self-reported diabetes, or self-reported use of antidiabetic medication.

### 2.5 Statistical analyses

For statistical analyses, we used Stata/SE 17.0 (StataCorp, College Station, Texas). A two-sided P-value <0.05 was considered statistically significant. To test for genotyping or population sampling errors (12), a Chi-squared test was used to evaluate Hardy–Weinberg equilibrium. Baseline characteristics at the day of enrollment were divided according to smoking status (never, former, current) and *CHRNA3* genotype (CC/CT/TT) and compared using Kruskal-Wallis test.

In observational analyses, risk of moderate to severe psoriasis according to smoking was assessed using Cox proportional hazard regression with age as underlying timescale (age-adjusted) and entry at study examination date (left truncation). Individuals with moderate to severe psoriasis prior to baseline (N=592) were excluded from the prospective analyses and individuals were followed until death (N=10,762), emigration (N=447), or end of follow-up which was 13^th^ of December 2018. Risk was assessed in a model only adjusted for age and sex and in a multivariable adjusted model including age (timescale), sex, BMI category, hypertension (yes/no), dyslipidemia (yes/no), high alcohol consumption (yes/no), type 2 diabetes (yes/no), low physical activity (yes/no), and low education level (yes/no). Sex and age-adjusted linear regression analysis was used to determine the increase in pack-years per *CHRNA3* T-allele. In supplementary analyses, the association between smoking and moderate to severe psoriasis was estimated using logistic regression. Because genes are present from birth and therefore not influenced by confounding, we included all psoriasis cases from January 1977 to December 2018 in the genetic analyses estimating the risk of moderate to severe psoriasis as a function of *CHRNA3* genotype in a logistic regression model only adjusted for age and sex.

Data on covariates were 98.8% complete. We had full information on sex and age. We imputed missing values of covariates using multivariable regression for continuous variables and chained equation for categorical values based on age and sex. Analyses including only individuals with full information on covariates gave similar results to those reported.

## 3 Results

Of the 105,912 participants included, we identified 1,240 individuals with moderate to severe psoriasis of whom 592 had psoriasis before examination date and 648 developed psoriasis during follow-up. In prospective observational analyzes, median follow-up was 9 years (interquartile range 7-12). Baseline characteristics are shown in **Table 1**. Compared to never smokers, former and current smokers were older, less likely to be women, and had a higher alcohol consumption. Current smokers had a lower level of education and physical activity and a higher prevalence of dyslipidemia. Former smokers had a higher prevalence of type 2 diabetes and hypertension. There were no differences in baseline characteristics according to *CHRNA3* genotype after correction of multiple comparisons. We found no sign of genotyping or population sampling errors (Hardy-Weinberg equilibrium P-value = 0.84).

**Table 1:** Baseline characteristics at the day of enrollment according to smoking status (upper section) and genotype (lower section) in individuals from the Copenhagen General Population Study.

	Smoking status			
	Never smokers N = 45,435 (43%)	Former smokers N = 42,464 (40%)	Current smokers N = 18,013 (17%)	*P* for comparison
Age, years	56 (46-66)	60 (51-69)	57 (48-65)	1 × 10^-300^
Women, N (%)	26,829 (59)	22,056 (52)	9,401 (52)	2 × 10^-113^
Body mass index, kg/m^2^	25 (23-28)	26 (24-29)	25 (23-28)	7 × 10^-153^
Hypertension, N (%)	22,129 (52)	23,232 (58)	9,053 (53)	6 × 10^-70^
Dyslipidemia, N (%)	32,661 (72)	30,965 (73)	13,734 (76)	6 × 10^-27^
Type 2 diabetes, N (%)	1,543 (3.4)	2,074 (4.9)	788 (4.4)	9 × 10^-28^
Alcohol consumption, units/week	84 (36-144)	120 (48-204)	108 (48-228)	1 × 10^-300^
Low physical activity, N (%)	2,268 (5.0)	2,270 (5.4)	1,992 (11.2)	9 × 10^-199^
Low education level, N (%)	22,846 (50)	22,499 (53)	12,339 (69)	1 × 10^-300^
	*CHRNA3* genotype			
	Non-carriers C/C N = 47,334 (45%)	Heterozygotes C/T N = 46,169 (44%)	Homozygotes TT N = 11,291 (11%)	*P* for comparison
Age, years	58 (48-67)	58 (48-67)	58 (48-67)	0.47
Women, N (%)	26,010 (55)	25,446 (55)	6,186 (55)	0.81
Body mass index, kg/m^2^	26 (23-28)	26 (23-28)	26 (23-28)	0.01
Hypertension, N (%)	24,319 (55)	23,665 (54)	5,846 (55)	0.44
Dyslipidemia, N (%)	34,520 (73)	33,820 (73)	8,274 (73)	0.50
Type 2 diabetes, N (%)	2,043 (4.3)	1,837 (4.0)	462 (4.1)	0.03
Alcohol consumption, units/week	96 (48-180)	96 (48-180)	96 (48-180)	0.60
Low physical activity, N (%)	2,842 (6.1)	2,893 (6.1)	707 (6.3)	0.20
Low education level, N (%)	25,536 (54)	25,103 (55)	6,038 (54)	0.18

Continuous variables are presented as medians (interquartile range) and categorical variables as N (%).

### 3.1 Observational analyses

**Figure 2:**
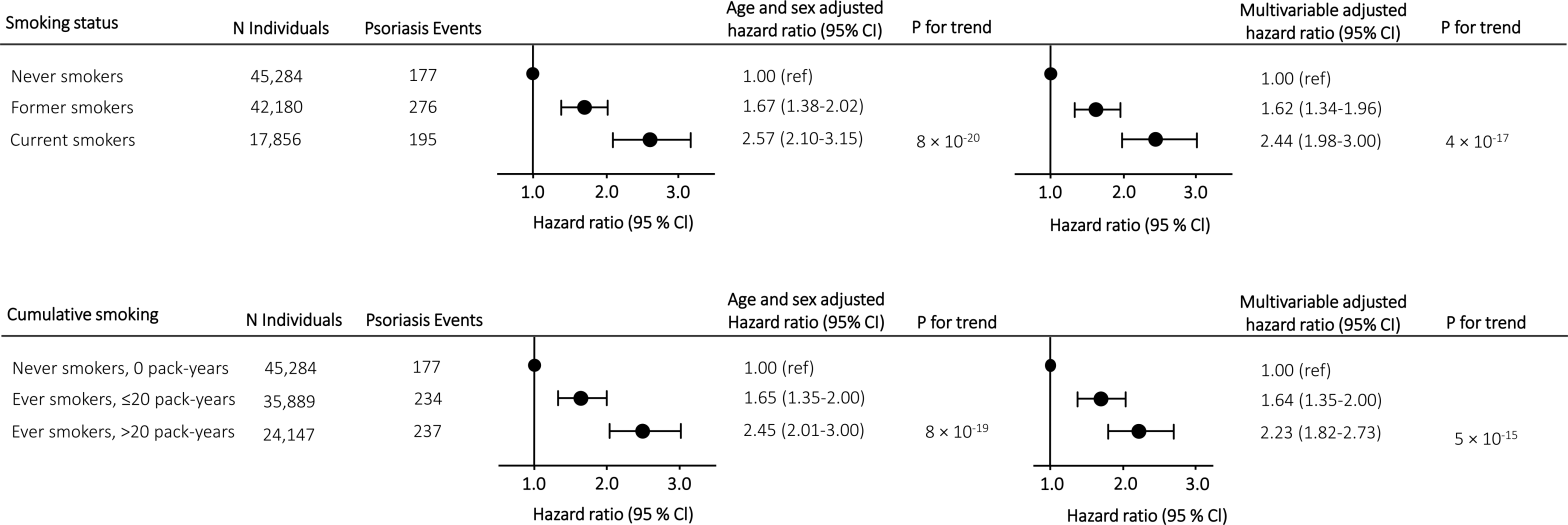
Risk of moderate to severe psoriasis according to smoking status and cumulative smoking in prospective analyses in individuals from the Copenhagen General Population Study. Hazard ratios were multivariable adjusted for age (underlying timescale), sex, BMI, hypertension, dyslipidemia, high alcohol consumption, type 2 diabetes, low physical activity, and low education level. Individuals diagnosed with moderate to severe psoriasis before examination date were excluded from the analyzes. Abbreviations: N, number.

In prospective analyses, we found a stepwise increase in risk of moderate to severe psoriasis according to smoking status with a multivariable adjusted hazard ratio of 1.62 (1.34-1.96) in former smokers and 2.44 (1.98-3.00) in current smokers compared to never smokers (**Figure 2**, upper panel). Risk of moderate to severe psoriasis according to cumulative smoking also increased in a stepwise matter with a multivariable adjusted hazard ratio of 1.64 (1.35-2.00) in ever smokers with ≤20 pack-years and 2.23 (1.82-2.73) in ever smokers with >20 pack-years compared to never smokers (**Figure 2**, lower panel). Risk estimates attenuated only slightly in multivariable adjusted analyses compared to analyses only adjusted for age and sex. Similar results were seen in cross-sectional analyses (compare **Figure 2** with **Supplementary Figure 1**).

Using a more limited definition of psoriasis, results were similar in both cross-sectional (compare **Figure 2** with **Supplementary Figure 2**) and prospective analyses (compare **Figure 2** with **Supplementary Figure 3**).

### 3.2 Genetic analyses

**Figure 3:**
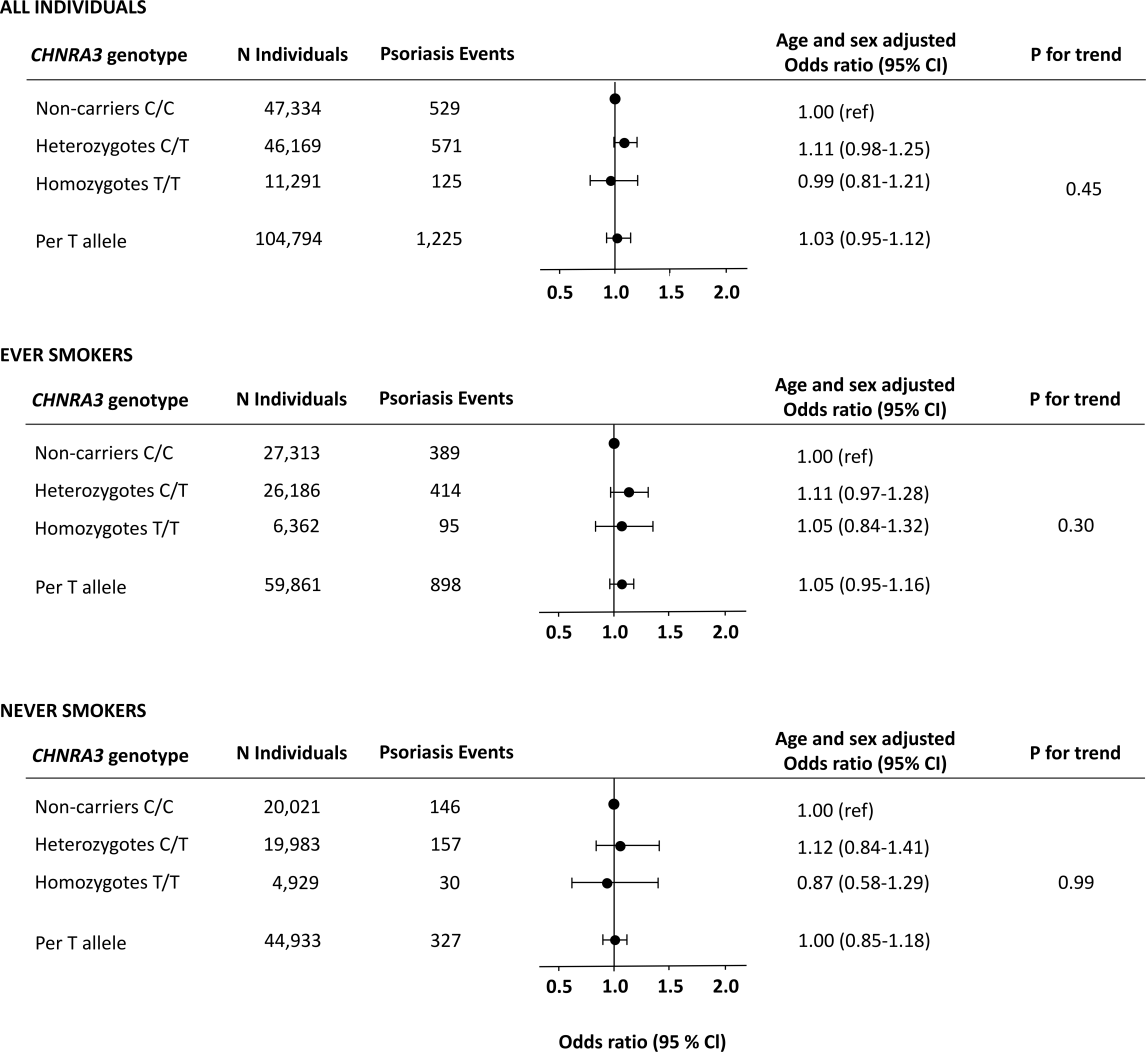
Risk of moderate to severe psoriasis as a function of the *CHRNA3* rs1051730 genotype in individuals from the Copenhagen General Population Study. Analyses were adjusted for age and sex. Upper panel: All individuals. Middle panel: Never smokers. Lower panel: Ever smokers.

Among the 104,794 individuals included in the genetic analyses, 47,334 were non-carriers (C/C), 46,169 were heterozygotes (C/T), and 11,291 were homozygotes (T/T). As anticipated, the *CHRNA3* genotype was associated with increased lifelong cumulative smoking. In concordance with previous literature, we found an increase of +1.6 pack-years per T-allele (P for trend=2 × 10^-40^) (**Supplementary Figure 4**). However, we did not find an increased risk of moderate to severe psoriasis according to *CHRNA3* genotype in analyses of all individuals or in analyses stratified by smoking status with an odds ratio for psoriasis of 1.05 (0.95-1.16) per *CHRNA3* rs1051730 T-allele in ever smokers (**Figure 3**). In analyses of both ever and never smokers, the age and sex adjusted hazard ratio of psoriasis was 1.11 (0.98-1.25) in heterozygotes (C/T) and 0.99 (0.81-1.21) in homozygotes (T/T) compared to non-carriers (C/C) (*P* for trend = 0.45) and results were similar in analyzes of only ever smokers and never smokers. In sensitivity analyses, using the more limited definition of psoriasis, results were likewise similar (compare **Figure 3** with **Supplementary Figure 5**).

## 4 Discussion

**Figure 4:**
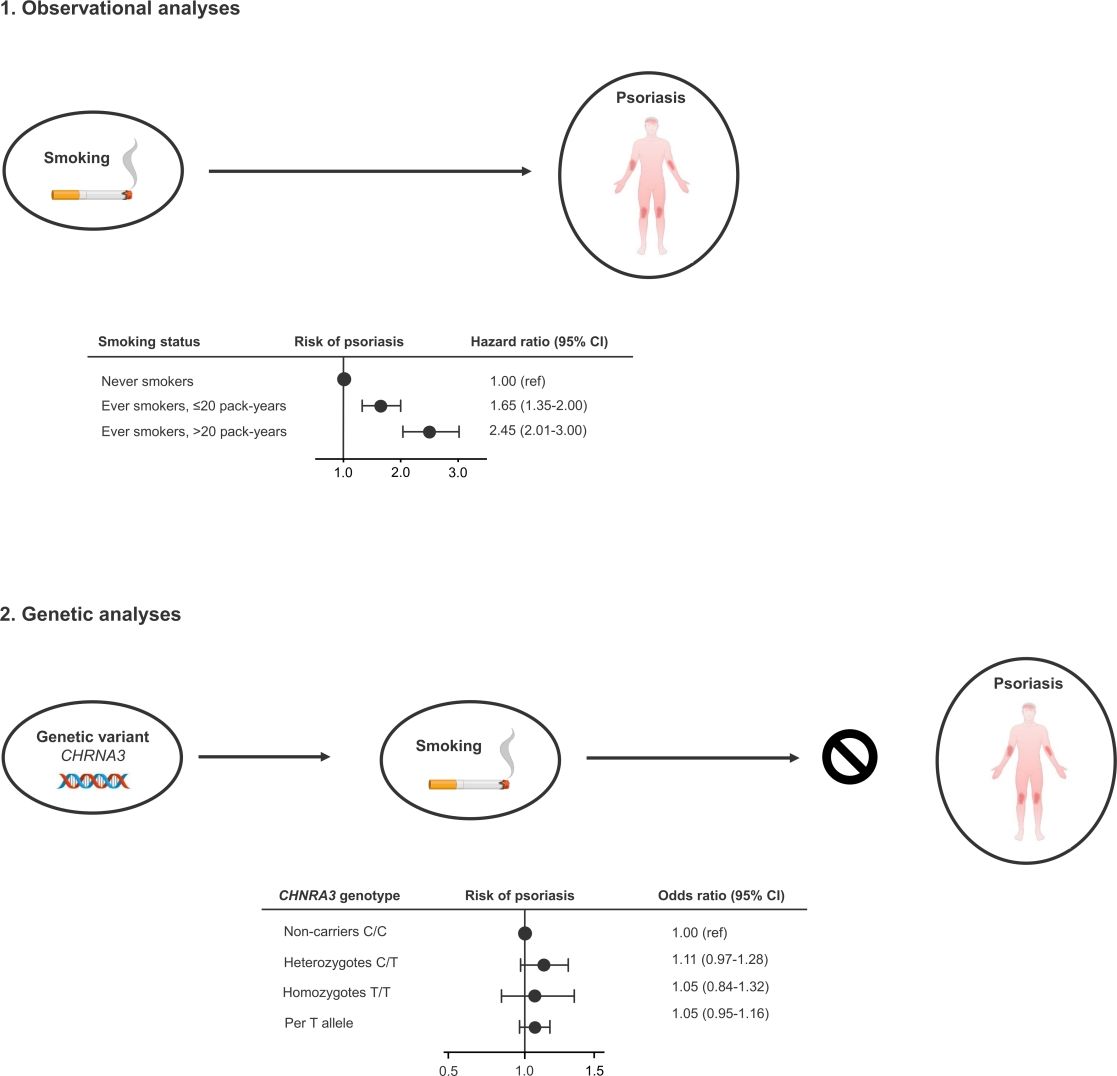
Take-home figure. Observationally, ever smokers (former and current smokers) have increased risk of developing moderate to severe psoriasis (1). However, using the genetic variant *CHRNA3* rs1051730 as a robust proxy for lifelong high cumulative smoking, we do not find an increased risk of moderate to severe psoriasis, indicating that smoking is an observational but not causal risk factors for moderate to severe psoriasis (2).

In this study using both observational and genetic data from more than 100,000 individuals from the adult Danish general population, we found a 2.5-fold higher risk of moderate to severe psoriasis in current smokers compared to never smokers. However, using the genetic variant *CHRNA3* rs1051730 as a proxy for high lifelong cumulative smoking, we did not find indications of a causal relationship between smoking and psoriasis (**Figure 4**).

Smoking could potentially be linked to psoriasis through several pathogenic pathways (26). Smoking causes oxidative stress which produces free radicals and induces the production of several proinflammatory cytokines involved in the pathogenesis of psoriasis (27). Additionally, smoking seems to modify the expression of several genes that confer increased risk of psoriasis such as *HLA-Cw6*, HLA-DQA1*0201, and *CYP1A1* (26). Moreover, results from epidemiological studies suggest an association between smoking and psoriasis. In the 80s and 90s, the first studies reporting associations between smoking and psoriasis were published (28–31) and subsequently, several observational studies (especially case-control and cross-sectional studies) found that patients with psoriasis smoke more than the general population, and that smoking is associated with more severe psoriasis (5). Furthermore, prospective studies have reported an increased risk of incident psoriasis among current smokers compared to never smokers (6–8, 10) and Groot et al. reported increased risk of pediatric psoriasis in children of mothers who smoked during pregnancy, including increased risk per each additional 5 cigarettes smoked daily, indicating a dose-response relationship (11).

The above-mentioned findings from observational studies could reflect a causal relationship between smoking and psoriasis; however, the association could also be explained by residual/unmeasured confounding or reverse causation. Smoking is associated with various lifestyle and socioeconomic factors (32), including comorbid diseases (33) that are also associated with psoriasis. Although observational studies try to adjust for several potential confounders, residual and/or unmeasured confounding can never fully be ruled out, and this is also the case with reverse causation. For example, the psychological burden of having psoriasis (34, 35) may lead to increased intensity of smoking to tackle life with a chronic skin disease and thus may explain the observed dose-response relationship in cross-sectional studies. Theoretically, the gold standard of proving causality is the randomized controlled trial; however, it is not feasible nor ethical to do this with smoking and psoriasis. The MR study mimics the randomized controlled trial by using genetic variants as robust proxies for modifiable exposures (**Figure 1**). The use of MR studies in medical science is increasing (13, 36), also in the field of psoriasis (37–42). Recently, two studies reported that smoking was a causal risk factor for psoriasis (38, 43), which we could not confirm. Wei et al (38) used summary statistics in their 2-sample MR, without access to individual-level data. Thus, smokers and never smokers could not be examined separately. As smoking can only be a cause among smokers, this might explain the different results. Zhao et al (43) used a genetic instrument, constituted of 126 genotypes, but with a F-value of 15 as opposed to 32 in this study indicating that their instrument is less strong. Furthermore, our instrument is believed to have a rather clear mode of action: the genetic variant *CHRNA3* rs1051730 is found in the nicotinic acetylcholine receptor gene cluster CHRNA5-A3-B4 on chromosome 15, which codes for the cerebral nicotine receptor. This leads to a variation in nicotine craving among smokers, explaining the strong association between the genetic variant and the trait (15, 20).

Strengths of this study includes the large study population and use of the unique Danish health registries. The Copenhagen General Population Study is a prospective cohort with a sample size of more than 100,000 from the general population with extensive amount of data on each participant, including genetic data free of confounding (21, 44). The Danish National Patient Registry is one of the oldest national heath registries in the world, and includes all in- and outpatient visits at the Danish hospitals since 1977 (24). Nevertheless, some limitations should be discussed. First, we only included individuals of Danish descent to minimize risk of population stratification, possibly reducing the generalizability of our findings. Second, information on smoking was collected at the day of enrollment in the study and some participants might have quit smoking during follow-up but would be defined as current smokers in our analyses. Finally, we identified individuals with psoriasis using ICD-8 and ICD-10 codes, which mainly captures individuals with moderate to severe psoriasis (23). Individuals with mild psoriasis might therefore be in our “non-psoriatic population”, potentially diluting the observational association between smoking and psoriasis.

In conclusion, in this study using both observational and genetic data from more than 100,000 individuals from the adult Danish general population, we found that smoking is observationally associated with a 2.5-fold higher risk of moderate to severe psoriasis in prospective analyses adjusted for multiple confounding factors. However, using the genetic variant *CHRNA3* rs1051730, as an instrument for high lifelong cumulative smoking, we did not find indications of a causal relationship between smoking and moderate to severe psoriasis (**Figure 4**). These results suggest that smoking is an independent, but not a causal risk factor for psoriasis. However, even though we do not find a causal association between smoking and moderate to severe psoriasis, smoking restriction and cessation remain important to minimize risk of smoking-related comorbidities.

## Data availability statement

The datasets presented in this article are not readily available because data from the Copenhagen General Population Study are subject to protection from the national Danish Data Protection Agency and we are not allowed to share the data ourselves. However, interested researchers can contact members of the steering committee to request data access. Additional data are available upon request and requests may be made to the corresponding author. Requests to access the datasets should be directed to charlotte.sigrid.erika.naeslund.koch@regionh.dk.

## Ethics statement

The studies involving human participants were reviewed and approved by the Danish ethical committees (H-KF-01-144/01). The patients/participants provided their written informed consent to participate in this study.

## Author contributions

All authors designed the study. CN-K and SV-K did the statistical analyses. CN-K wrote the first draft of the manuscript and created figures and tables. All authors read, revised, and accepted the final article.
