# Supplementary material for: Smoking is an independent but not a causal risk factor for moderate to severe psoriasis: A Mendelian randomization study of 105,912 individuals
Source: Front Immunol. 2023 Feb 22;14:1119144. doi: 10.3389/fimmu.2023.1119144 (PMC9992829; doi:10.3389/fimmu.2023.1119144)
Supplement: Supplementary file 1 [file Presentation_1.pdf]

Supplementary Material

Smoking is an independent but not a causal risk factor for moderate to severe psoriasis: a Mendelian randomization study of 105,912 individuals

Charlotte Näslund-Koch*, Signe Vedel-Krogh, Stig Egil Bojesen, Lone Skov

*** Correspondence:**
charlotte.sigrid.erika.naeslund.koch@regionh.dk

1 Supplementary Figures

The following figures are attached in this document:
- Supplementary Figure 1
- Supplementary Figure 2
- Supplementary Figure 3
- Supplementary Figure 4
- Supplementary Figure 5

1.1 Supplementary Figure 1

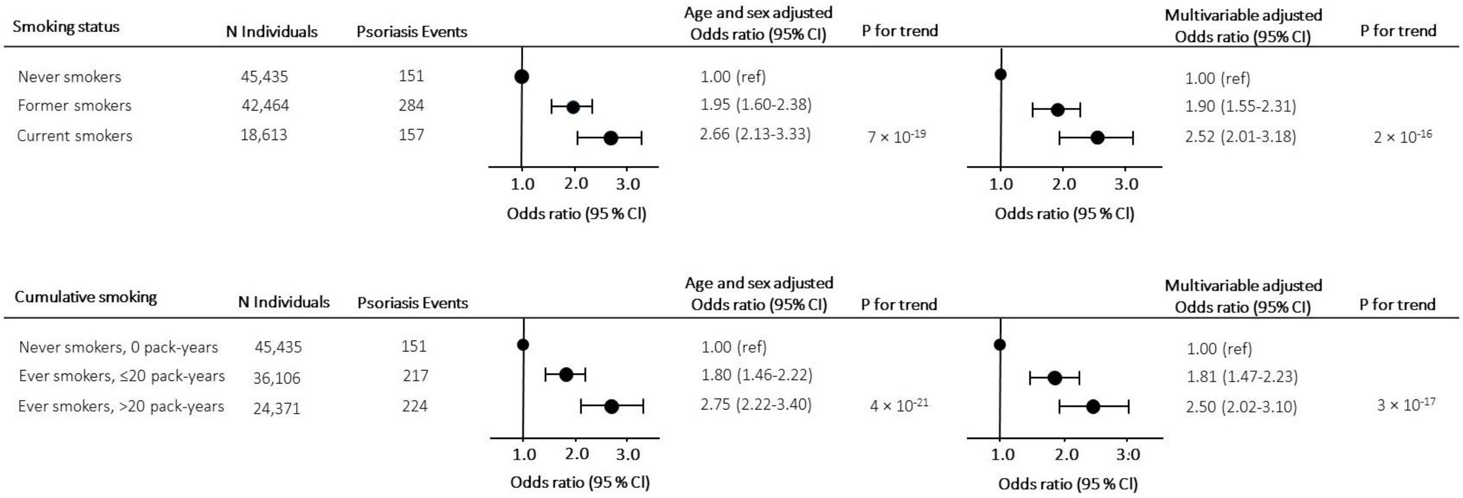

Supplementary Figure 1. Risk of moderate to severe psoriasis according to smoking status and cumulative smoking in cross-sectional analyses in individuals from the Copenhagen General Population Study. Odds ratios were multivariable adjusted for age, sex, body mass index, hypertension, dyslipidemia, high alcohol consumption, type 2 diabetes, low physical activity, and low education level.

Abbreviations: CI, Confidence Interval. N, number

1.2 Supplementary Figure 2

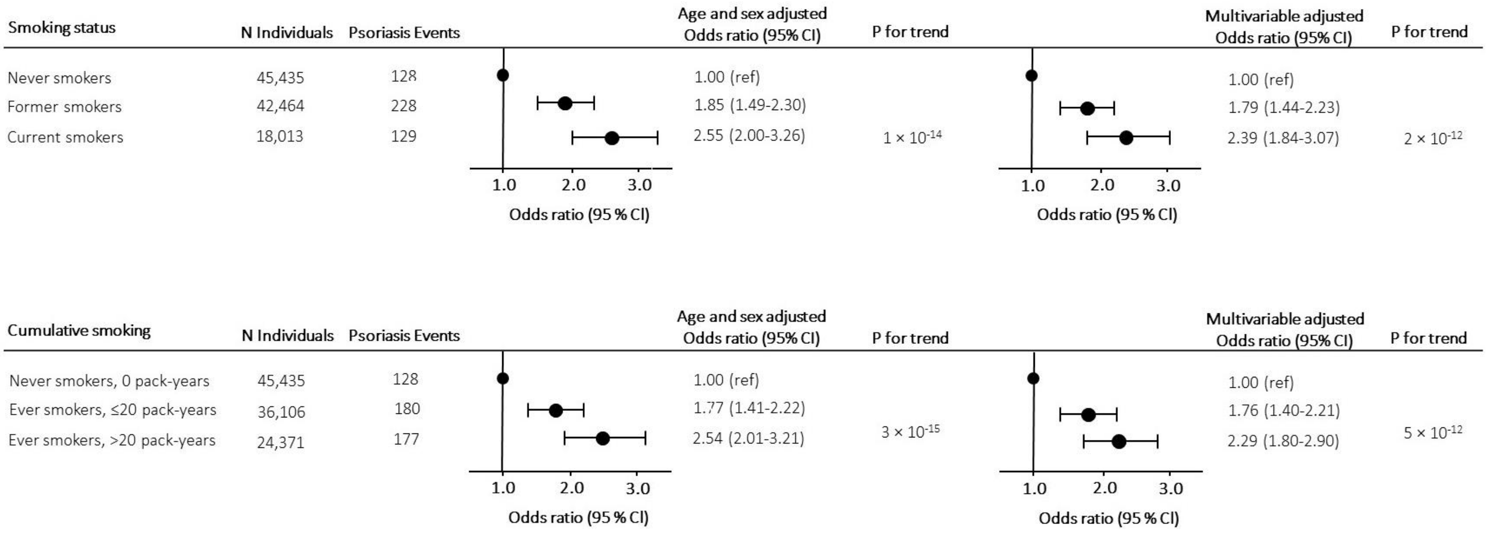

Supplementary Figure 2. Risk of moderate to severe psoriasis (using *only* ICD-8; 696.19 and ICD-10; L40.0 and L40.9) according to smoking status and cumulative smoking in cross-sectional analyses in individuals from the Copenhagen General Population Study. Odds ratios were multivariable adjusted for age, sex, body mass index, hypertension, dyslipidemia, high alcohol consumption, type 2 diabetes, low physical activity, and low education level.

Abbreviations: CI, Confidence Interval. N, number

1.3 Supplementary Figure 3

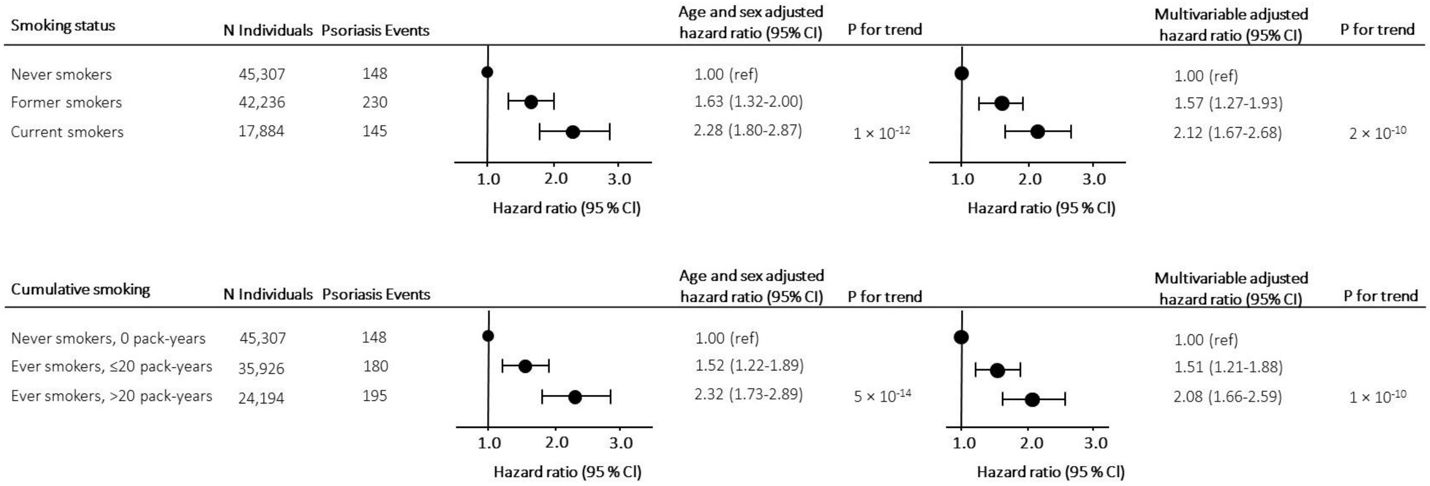

Supplementary Figure 3. Risk of moderate to severe psoriasis (using *only* ICD-8; 696.19 and ICD-10; L40.0 and L40.9) according to smoking status and cumulative smoking in prospective analyses in individuals from the Copenhagen General Population Study. Hazard ratios were multivariable adjusted for age, sex, body mass index, hypertension, dyslipidemia, high alcohol consumption, type 2 diabetes, low physical activity, and low education level.

Abbreviations: CI, Confidence Interval. N, number

1.4 Supplementary Figure 4

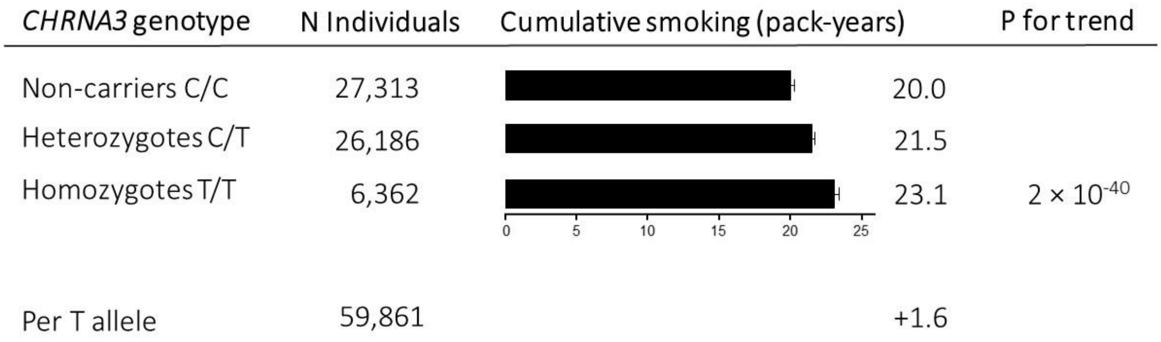

Supplementary Figure 4. Cumulative smoking measured in pack-years in former and current smokers according to *CHRNA3* rs1051730 genotype. Results are means with 95% Confidence Intervals. P for trend is from a non-parametric trend test.

Abbreviations: N, number

1.5 Supplementary Figure 5

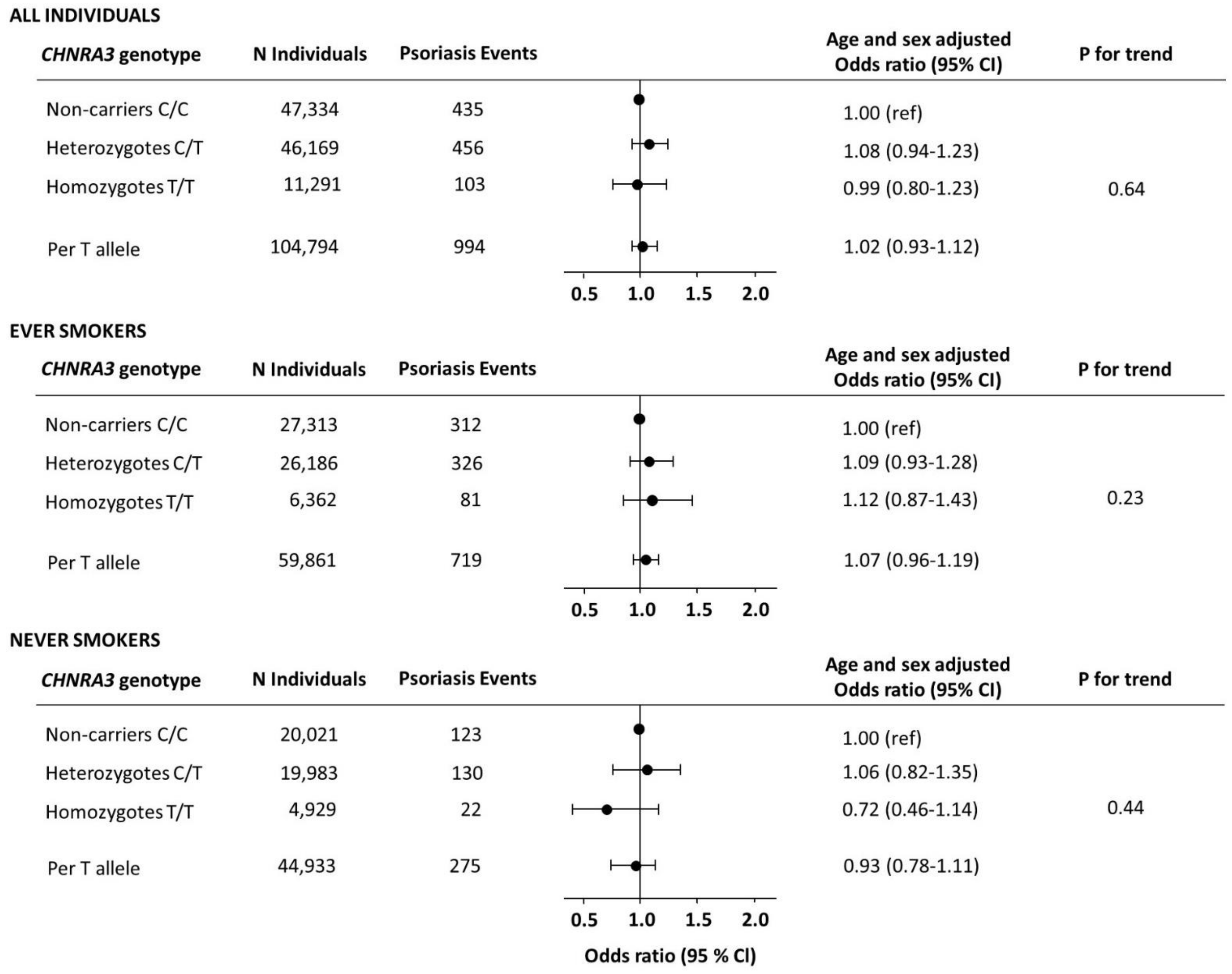

Supplementary Figure 5. Risk of moderate to severe psoriasis (using *only* ICD-8; 696.19 and ICD-10; L40.0 and L40.9) as a function of *CHRNA3* genotype in individuals from the Copenhagen General Population Study. Analyses were only adjusted for age and sex.
Upper panel: All individuals.
Middle panel: Never smokers.
Lower panel: Ever smokers

Abbreviations: CI, Confidence Interval. N, number
